# The likelihood ratio and frequency of DQ2/DQ8 haplotypes in Iranian patients with celiac disease 

**Published:** 2016

**Authors:** Asghar Khosravi, Masoume Mansouri, Mohammad Rostami-Nejad, Bijan Shahbazkhani, Golnaz Ekhlasi, Ebrahim Kalantari

**Affiliations:** 1 *Digestive Disease Research Center, Digestive Disease Research Institute, Tehran University of Medical Sciences, Shariati Hospital, Tehran, Iran*; 2 *Health Center of Tarbiat Modares University, Tehran, Iran*; 3 *Gastroenterology and Liver Diseases Research Center, Research Institute for Gastroenterology and Liver Diseases, Shahid Beheshti University of Medical Sciences,Tehran, Iran*; 4 *Gholhak Medical Laboratory. Tehran, Iran*

**Keywords:** Celiac disease, HLA DQ2, HLA DQ8, HLA typing, Likelihood ratio, Iranian population

## Abstract

**Aim:**

The aim of this study was to evaluate the likelihood ratio and frequency of DQ2 and DQ8 in Iranian patients with celiac disease (CD).

**Background:**

The HLA DQ2 and HLA DQ8 are the important mediators in the development of celiac disease. A few studies evaluated the frequency of HLA DQ2 and HLA DQ8 haplotypes among the Iranian population with low sample size.

**Patients and methods:**

In this cross-sectional study, to predict HLA–DQ2 and DQ8 haplotypes, 141(73 male, 78 female) confirmed CD patients compared to 151 healthy controls were enrolled into this study during 2013-2014. HLA DQ2/ DQ8 haplotypes was determined in cases and controls using PCR-SSP technique.

**Results:**

DQ2 and DQ8 were positive in 80% (n=111) and 49% (n= 69) of CD patients and 36% (n=61) and 13% (n=21) of control group respectively. Moreover, 32% (n=45) of CD patients and 5.3% (n=8) of the control group were carrier of both haplotypes. In the case group about one-third of patients (32.2%) were positive for carrying both DQ2 and DQ8 heterodimers while only 5.3% (n=8) of the control group were carrier. In addition, the positive likelihood ratio of DQ2 and DQ8 were 1.74 (CI: 1.4- 2.1), and 2.6 (CI: 1.8– 2.7), respectively.

**Conclusion:**

The result of this study showed that the frequency of DQ8 among our population is higher than those reported by European countries, but it is close to those founded in South America and Middle East. This result suggests that the higher prevalence of HLA DQ8 pattern in Iranian CD patients is similar to non-European patients.

## Introduction

 Celiac disease (CD) is a chronic immune-mediated disorder which is induced by the ingestion of gluten containing cereals in genetically predisposed subjects (1-4). It has been established that the HLA DQ2 and HLA DQ8 have a great role in the development of celiac disease (5-7). In addition, cells expressing DQ2 and DQ8 which show high affinity to gluten derived products, are present in the small intestinal mucosa of CD patients (8-10). Epidemiological studies showed that the frequency of DQ2-DQ8 among European populations with celiac disease is almost the same and DQ2 is predominant (more than 90%) (11-15). However, in non-European countries*, *different frequencies have been reported (16-19). The DQ8 frequency was estimated from 3 to 8% in different parts of Europe (12) and from 4.2 % in the USA to 43% in Argentina in non-European populations (16, 20). On the other hand, few studies evaluated the distribution of HLA DQ haplotypes in Iranian patients with CD (21). In the preliminary study, Rostami Nejad et al. reported that 97% of CD cases and 58% of controls were carriers of HLADQ2 and/or HLA DQ8 heterodimers, either in the homozygous or heterozygous state and afterward Zamani et al., confirmed these findings (22). 

 In the present study, we aimed to evaluate the frequency of HLADQ2 and/or HLA DQ8 among Iranian CD patients and compare DQ2 and DQ8 likelihood ratio in biopsy confirmed CD patients and healthy controls living in Tehran (the capital city of Iran). 

## Patients and Methods

**Celiac group**

Patients with confirmed CD (n = 140, 93 females and 47 males, mean age 38.37 years) were referred to the Digestive Disease Research Center, Digestive Disease Research Institute, Tehran University of Medical Sciences. The CD patients were recruited over a period of 12-months from 2013 to 2014. The sample size was determined based on the previous study in Iran by Rostami-Nejad et al. (21) who showed the prevalence of homozygote HLA DQ2 in patients with Celiac disease is 27.1% and in the control group is 3.2%. Demographics, clinical and disease history were collected by checklist. Informed consent was obtained from each patient or patient’s guardian prior to the study enrollment. According to the Marsh classification (Marsh I-III), all patients had positive tTGA and/or EMA antibodies and histology (6). The study was approved by the ethics review board of Digestive Disease Research Center, Digestive Disease Research Institute, Tehran University of Medical Sciences and complied with the Helsinki declaration.

**Control group**

A total of 151 healthy controls with no history of CD, GI disease, cancer or autoimmune diseases were selected from the National Blood Transfusion Organization of Iran. Then, healthy controls (78 females and 73 males, mean age 40.43 years) were matched by age. 

**Sample collection and analysis**

Peripheral blood was collected in ethylene-diamino-tetra-acetic (EDTA) tubes and stored at -20^◦^C till further analysis. Genomic DNA was extracted from the whole blood using QIAamp DNA Blood Mini Kit (Qiagen, Hilden, Germany) and polymerase chain reaction (PCR). Amplification with sequence specific primers (SSP) was used to detect DQA1*05, DQB1*02 and DQB1*0302 alleles using Olerup SSP™ PCR amplification kit (Genovision, Oslo). Samples were electrophoresed, stained with Sybr Green (Fluka Bio Chemica, Ronkonkoma, NY, USA), and photographed under the exposure to UV light (Bio-Rad). Carriers of DQA1*05 and DQB1*02 were marked HLA-DQ2-positive, and carriers of DQB1*0302 were marked HLA-DQ8-positive

**Statistical analysis**

Data were analyzed using STATA (Version 11, Stata Corp, College Station, TX, USA) and SPSS softwares (version 15, SPSS Inc, Chicago, Illinois, USA). Data were presented as number and percentage (%) for categorical variables. Cross tabulation was performed to obtain the frequency of each allele among CD patients and control group. Comparing the independent proportion between two groups was performed by Pearson’s chi square test. To find the relationship between two conditional variables such as celiac disease and gender, Cramer’s Index was used. The Odds ratio was used to determine the association between groups (case- control) status and gender. Also the likelihood ratio was used for assessing the value of the diagnostic tests. The P value <0.05 was considered statistically significant. 

## Results

Characteristics of patient and control groups are summarized in table 1. The ethnic diversity of the CD patients included Fars (25.5%), Turk (14.2%), Gilakis/Mazandaranis (2.8%), Kurd (14.2%), Lur (4.3%), Torkman (1.4%) and Baluch (0.7%). 

**Table 1:** Characteristics of patient and control groups

Group	Celiac group	Control group
Mean age(years)	38.37	40.43
Male	47(39.2%)	73(60.8%)
Female	93(59.4%)	78(45.6%)

**Figure 1:**
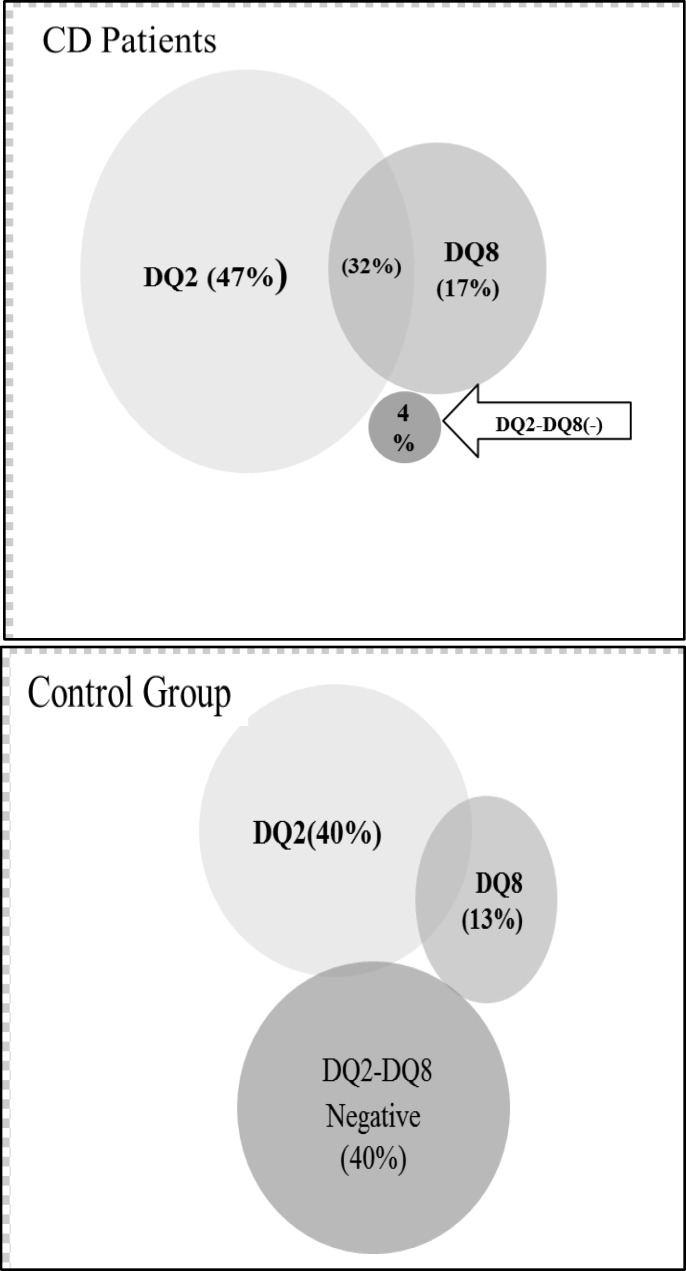
The DQ2 and DQ8 Frequency in CD patients and control group

HLA DQ2 and DQ8 were carried by 80% and 49% of CD patients, while the prevalence of these heterodimers in the control group was 36% and 25%, respectively. The frequency distribution of HLA DQ2 and DQ8 in patients with CD and control group is shown in figure 1. The comparison of HLA DQ frequency in CD patients and the control group is demonstrated in table 2. 

**Table 2:** The comparison of HLA DQ frequency in CD patients and control group

HLA classification	Celiac group (N=140)	Control group (N=151)	p-value
DQ2	66(47.1%)	1(40.4%)	0.001
DQ8	24(17.1 %)	21(13.9%)	
DQ2/DQ8 Pos	45(32.1%)	8(5.30%)	
DQ2/DQ8 Neg	5(3.6%)	61(40.4%)	

Table 2 shows that, a significant difference has been detected between two groups in terms of HLA DQ8 and HLA DQ2 (P<0.001). Based on this description, DQ2 and DQ8 haplotypes were

presented together in 96.4% of CD patients and 58.7% of controls. Cross tabulation analysis showed that, in a celiac group about one-third of patients (32.1%) were positive for carrying both DQ2 and DQ8 heterodimers. 

A positive likelihood ratio of DQ2 and DQ8 were 1.74 (CI: 1.4- 2.1), and 2.6 (CI: 1.8– 2.7), respectively. Similarly, the positive likelihood ratio of DQ2 and DQ8 together were 7.7 (CI: 4.0-15.0). The positive and negative likelihood ratio (LR) of HLA DQ haplotypes summarized in table 3. 

The odds ratio for females with celiac disease was 1.19 (95% CI: 0.88- 1.61) in compared with female without CD. For males, the odds ratio was 0.64 (95% CI: 0.45-0.93). 

**Table 3:** The positive and negative likelihood ratio (LR) of HLA DQ haplotypes

	Positive LR	95% CI	Negative LR	95% CI
HLA DQ2	1.74	1.43-2.11	0.38	0.26-0.54
HLA DQ8	2.57	1.78-3.71	0.63	0.52-0.75
HLA DQ2 -DQ8 Heterodimers	7.8	4.02-15.00	0.11	0.05-0.26

**Table 4:** The risk of celiac disease in male and female

sex	Celiac group	Control group	odds	95%CI	p-value
male	47(39.2%)	73(60.8%)	0.64	0.45-0.93	P<0.01
female	93(59.4%)	78(45.6%)	1.19	0.88-1.61	
total	140	151			

The prevalence of celiac was statistically significant different by sex (F/M=2; P<0.01). The risk of celiac disease in male and female is shown in table 4. Based on DQ2-DQ8 variables, the statistical power of this study (1-β) is calculated 99%. Table 5 showed the distribution of HLA genotype based on the ethnicity in Iranian patients with CD. 

## Discussion

The results of this study showed that 79.3% and 49.3% of CD patients were positive for carrying both DQ2 and DQ8 haplotypes, respectively. Moreover, 32% of CD patients shared both haplotypes (Figure 1). Previous studies estimated that the prevalence of celiac disease in Iranian population is about 1% (23- 25). However, a few data are available on the frequency of HLA-related CD predisposing genes in Iranian CD patients. 

Our result is compatible with worldwide studies, which showed that the DQ2 and DQ8 are the most important haplotypes in celiac disease (16-19). However, the frequency of these alleles is in diverse in different populations. 

Results from European studies showed that around 86 to 93% of CD patients are positive for carrying DQ2 variants, while the minority of them is DQ8 positive (26-28). However, two Italian studies reported that 80.8% of CD patients were positive for carrying HLA-DQ2 variants (5,29). Therefore, the frequency of HLD-DQ2 in our study is different from those reported by Northern European (Scandinavian probands, UK) studies and is similar to those observed in Southern European populations (France and Italy) (27-28).

The frequency of HLA-DQ8 in our celiac patients was 49.3%. In consistent to our study, the high frequency of DQ8 has also been reported in American Indians (25.3%), South America (28.3%), Middle East (22%) and Bushman (30%) (30). In this regard, the highest DQ8 frequency has been reported by a study conducted in Argentina (43.2%) (20).

In the previous studies in Iran, the same frequency of CD-related HLA-DQ heterodimers for celiac patients was reported. In the first study by Rostami-Nejad et al. (21), 83.03% of cases and 35.09% of controls were positive for carrying HLA-DQ2 heterodimer. Our study also expresses similar frequency of HLA-DQ2 genotype. In addition, the present study confirms the previous Iranian findings which show that the frequency of HLA DQ8 in the Iranian CD patients is high (21). 

**Table 5:** Distribution of HLA genotype based on ethnicity in Iranian CD patients

HLA DQ classification					
Ethnicity	DQX/DQX	DQ2/DQ8	DQ8/DQX	DQ2/DQX	TOTAL
Fars	1(0.7%)	16(11.3)	6(4.3%)	13(9.2%)	36(25.5%)
Kurd	0	6(4.3%)	6(4.3%)	8(5.7%)	20(14.2%)
Lur	0	2(1.4%)	1(0.7%)	3(2.1%)	6(4.3%)
Turk	0	6(4.3%)	4(2.8%)	10(7.1%)	20(14.2%)
Baluch	0	1(0.7%)	0	0	1(0.7%)
Torkman	0	0	0	2(1.4%)	2(1.4%
Gilakis	0	1(0.7%)	0	0	1(0.7%)
Mazandaranis	0	0	1(0.7%)	2(1.4%)	3(2.1%)
Afghani	0	1(0.7%)	1(0.7%)	0	2(1.4%)
Unknown	4(2.8%)	12(8.5%)	5(3.5%)	29(20.6%)	50(35.5%)
Total	5(3.5%)	45(31.9%)	24(17.0%)	67(47.5%)	141(100%)

 Our study population was originated from Tehran, the capital of Iran and populated by people from different origin and ethnicity. Different ethnicities, including Turk, Gilakis/Mazandaranis, Kurd, Lur, and Baluch’s are living in Tehran, but the population of Persian ethnicity is predominant. Many studies support the role of ethnicity and genetic background as an important factor in the distribution of CD-related HLA-DQ alleles (31). Thus, the higher frequency of DQ8 reported by this study may be due to the ethnic diversity of our study patients. 

We also used likelihood ratio (LR) as a measure of diagnostic accuracy to evaluate the strength of HLADQ typing for diagnosis of celiac disease in our clinical setting (32). Our result showed that positive and the negative LR for both DQ2-DQ8 genetic tests were 7.8 and 0.11, respectively. According to Guyatt et al. (33) a positive LR greater than ~2 or a negative LR less than ~0.5 is indicative of a useful test. Thus, DQ2 and DQ8 together are useful genetic markers for confirming (positive LR 7.76, 95% CI 1.92 to 16.10) and excluding (negative LR 0.11, 95% CI 0.06 to 0.37) the celiac diagnosis in our clinical setting (Table 3). 

With regard to gender differences, our findings showed women have a slightly greater risk of developing the celiac disease than men. Additionally, an increased prevalence of CD among women has been observed. These findings are also consistent with studies, which showed that CD occurs more often in female than in male with a gender ratio of about 2:1 (34-36).

The main limitation of this survey is that the ethnicity of 50 out of 140 patients are not exactly specified.T he second limitation was that, apart from DQA1*05 and DQB1*02, the other predisposing HLA-DQ2 alleles have not been identified in this project. These undetected alleles were classified as DQX haplotype in this study. 

Finally, we concluded that the frequency of DQ8 among Iranian CD patients is much higher than in those reported by European population and is in agreement with findings in South America and Middle East. According to differences and similarities of findings in different regions, performing a multicenter study may be useful to draw a map of HLA DQ typing. Additionally, the high likelihood ratio (LR>7) of both HLA DQ2 and DQ8 indicated that these genetic markers could be used to rule out the celiac disease in Iranian population.

## References

[1] Schuppan D (2000). Current concepts of celiac disease pathogenesis. Gastroenterology.

[2] Petronzelli F, Bonamico M (1997). Genetic contribution of the HLA region to the familial clustering of coeliac disease. Ann Hum Genet.

[3] Schuppan D (2000). Current concepts of celiac disease pathogenesis. Gastroenterology.

[4] Rostami Nejad M, Rostami K, Yamaoka Y, Mashayekhi R, Molaei M, Dabiri H (2011). Clinical and histological presentation of Helicobacter pylori and gluten related gastroenteropathy. Arch Iran Med.

[5] Megiorni F, Mora B, Bonamico M, Barbato M, Nenna R, Maiella G (2009). HLA-DQ and risk gradient for celiac disease. Hum Immunol.

[6] Wroblova K, Kolorz M, Pav I, Horakova Z, Filipova F, Bartos M (2014). Frequencies of HLA-DQ2 and HLA-DQ8 haplotypes in Czech and Slovak coeliac patients and the healthy population. Acta Biochemica Polinica (ABP).

[7] Kagnoff MF (2007). Celiac disease: pathogenesis of a model immune genetic disease. J Clin Invest.

[8] Molberg O, McAdam SN, Korner R, Quarsten H, Kristiansen C, Madsen L (1998). Tissue transglutaminase selectively modifies gliadin peptides that are recognized by gut derived T cells in celiac disease. Nat Med.

[9] Van de Wal Y, Kooy Y, Van VP, Pena S, Mearin L, Papadopoulos G, Koning F (1998). Selective deamidation by tissue transglutaminase strongly enhances gliadin-specific T cell reactivity. J Immunol.

[10] Lundin KEA, Scott H, Hansen T, Paulsen G, Halstensen TS, Fausa O (1993). Gliadin-specific, HLA DQ restrict T cells isolated from the small intestinal mucosa of celiac disease patients. J Exp Med.

[11] Sollid LM (2002). Coeliac disease: dissecting a complex inflammatory disorder. Nat Rev Immunol.

[12] Polvi A, Arranz E, Fernandez-Arquero M, Collin P, Mäki M, Sanz A (1998). HLA-DQ2-negative celiac disease in Finland and Spain. Human Immunol.

[13] Kagnoff MF (2007). Celiac disease: pathogenesis of a model immunogenetic disease. J Clin Invest.

[14] Megiorni F, Mora B, Bonamico M, Barbato M, Montuori M, Viola F (2008). HLA-DQ and susceptibility to celiac disease: evidence for gender differences and parent-of-origin effects. Am J Gastroenterol.

[15] Romanos J, van Diemen CC, Nolte IM, Trynka G, Zhernakova A, Fu J (2009). Analysis of HLA and non-HLA alleles can identify individuals at high risk for celiac disease. Gastroenterology.

[16] Johnson TC, Diamond B, Memeo L, Negulescu H, Hovhanissyan Z, Verkarre (2004). Relationship of HLA-DQ8 and severity of celiac disease: comparation of New York and Parisian cohorts. Clin Gastroenterol Hepatol.

[17] Butterworth JR, Iqbal TH, Cooper BT (2005). Coeliac disease in South Asians residents in Britain: comparation with white Caucasian coeliac patients. Eur J Gastroenterol Hepatol.

[18] Cintado A, Sorell L, Galván JA, Martínez L, Castañeda C, Fragoso T (2006). HLA DQA1*0501 and DQB1*02 in Cuban celiac patients. Human Immunol.

[19] Araya M, Mondragon A, Perez-Bravo F, Roessler JL, Alarco´n T, Rios G (2000). Celiac disease in a Chilean population carrying Amerindians traits. J Pediatr Gastroenterol Nutr.

[20] Layrisse Z, Guedez Y, Domínguez E, Paz N, Montagnani S, Matos M (2001). Extended HLA haplotypes in a Carib Amerindian population: the Yucpa of the Perija Range. High frequency of DQ8 in the celiac population of Chaco province, Argentina. Hum Immunol.

[21] Rostami-Nejad M, Romanos J, Rostami R, Ganji A, Ehsani-Ardakani MJ, Bakhshipour AR (2014). Allele and haplotype frequencies for HLA-DQ in Iranian, celiac disease patients. World J Gastroenterol.

[22] Zamani F, Mohamadnejad M, Shakeri R, Amiri A, Najafi S, Alimohamadi SM (2008). Gluten sensitive enteropathy in patients with iron deficiency anemia of obscure origin. World J Gastroenterol.

[23] Shahbazkhani B, Malekzadeh R, Sotoudeh M, Moghadam KF, Farhadi M, Ansari R (2003). High prevalence of coeliac disease in apparently healthy Iranian blood donors. Eur J Gastroenterol Hepatol.

[24] Rostami-Nejad M, Villanacci V, Mashayakhi R, Molaei M, Bassotti G, Zojaji H (2009). Celiac disease and Hp infection association in Iran. Rev Esp Enferm Dig.

[25] Rostami-Nejad M, Rostami K, Pourhoseingholi MA, Nazemallhosseini Mojarad E, Habibi M, Dabiri H (2009). Atypical presentation is dominant and typical for coeliac disease. J Gastrointestin Liver Dis.

[26] Rostami K, Aldulaimi D, Holmes G, Johnson MW, Robert M, Srivastava A (2015). Microscopic enteritis: Bucharest consensus. World J Gastroenterol.

[27] Karell K, Louka AS, Moodie SJ, Ascher H, Clot F, Greco L (2003). HLA types in celiac disease patients not carrying the DQA1*05- DQB1*02 (DQ2) heterodimer: results from the European Genetics Cluster on Celiac Disease. Hum Immunol.

[28] Margaritte-Jeannin P, Babron MC, Bourgey M, Louka AS, Clot F, Percopo S (2004). HLA-DQ relative risks for coeliac disease in European populations: a study of the European Genetics Cluster on Coeliac Disease. Tissue Antigens.

[29] Piccini B, Vascotto M, Serracca L, Luddi A, Margollicci MA, Balestri P (2012). HLA-DQ typing in the diagnostic algorithm of celiac disease. Rev Esp Enferm Dig.

[30] Catassi C, Yachha SK, Fasano A, Troncone R, Branski D The global village of celiac disease. Frontiers in Celiac Disease.

[31] Alaridaa K, Harownb J, Di Pierroc MR, Dragoc S, Catassid C (2010). HLA-DQ2 and -DQ8 genotypes in celiac and healthy Libyan. Dig Liver Dis.

[32] Boyko EJ (1994). Ruling out or ruling in disease with the most sensitive or specific diagnostic test: short cut or wrong turn. Med Decis Making.

[33] Guyatt G, Rennie D, Meade M, Cook D (2008). User's Guides to the Medical Literature: Essentials of Evidence-based Clinical Practice.

[34] Kagnoff MF (2007). Celiac disease: pathogenesis of a model immunogenetic disease. J Clin Invest.

[35] Llorente-Alonso MJ, Fernandez-Acenero MJ, Sebastian M (2006). Gluten intolerance: sex and age-related features. Can J Gastroenterol.

[36] Megiorni F, Mora B, Bonamico M, Barbato M, Montuori M, Viola F (2008). HLA-DQ and susceptibility to celiac disease: evidence for gender differences and parent-of-origin effects. Am J Gastroenterol.

